# The impact of combined administration of ropivacaine and dexamethasone on postoperative analgesia in perianal surgery with pudendal nerve block under ultrasound guidance: a prospective randomized controlled study

**DOI:** 10.3389/fphar.2024.1366070

**Published:** 2024-06-27

**Authors:** Tao-Ran Yang, Dan Pu, Yan Cheng, Cheng-Xi Fan, Ya-Jun Hu, Ru-Rong Wang, Xue-Han Li

**Affiliations:** ^1^ Department of Anesthesiology, West China Hospital, Sichuan University, Chengdu, China; ^2^ The Research Units of West China (2018RU012)-Chinese Academy of Medical Sciences, West China Hospital, Sichuan University, Chengdu, China; ^3^ Lung Cancer Center, West China Hospital, Sichuan University, Chengdu, China; ^4^ Department of Anesthesiology, West China Hospital, Sichuan University/Chengdu Shang Jin Nan Fu Hospital, Chengdu, China

**Keywords:** perianal surgery, pudendal nerve block, perioperative pain management, dexamethasone, urinary retention

## Abstract

**Background:**

In recent years, severe pain after perianal surgery has seriously affected the prognosis of hospitalized patients. How to maximize the improvement of postoperative pain and perioperative comfort becomes particularly important.

**Methods:**

This study was a double-blind randomized controlled trial (Registration No.: ChiCTR2100048760, Registration Date: 16 July 2021, Link: www.chictr.org.cn/showproj.html?proj=130226), and patients were randomly divided into two groups: one group underwent postoperative 20 mL bilateral pudendal nerve block with 0.5% ropivacaine (P group), and the other group underwent postoperative 20 mL bilateral pudendal nerve block with 0.5% ropivacaine + 8 mg dexamethasone (PD group). The primary outcome was the incidence of moderate to severe pain at the first postoperative dressing change. Secondary outcomes included Quality of recovery-15 (QoR-15) score at 3 days after surgery, sleep quality, pain score at 3 days after surgery, and incidence of adverse events.

**Results:**

In the main outcome indicators, the incidence was 41.7% in the P group and 24.2% in the PD group (*p* = 0.01). The QoR-15 score and sleep quality in PD group were better than those in P group 2 days before surgery. The incidence of postoperative urinary retention was significantly decreased in PD group (*p* = 0.01).

**Conclusion:**

Local anesthesia with dexamethasone combined with pudendal nerve block after perianal surgery can reduce the incidence of moderate to severe pain during the first dressing change. This may be one of the approaches to multimodal analgesia after perianal surgery.

**Clinical Trial Registration:**

https://www.chictr.org.cn/, identifier ChiCTR2100048760.

## 1 Introduction

Perianal diseases refers to a series of conditions including mixed hemorrhoids, perianal abscess, anal fistula, and anal fissure (Jain et al., 2019). According to the epidemiological data from the United States, the prevalence rate of hemorrhoids alone is as high as 55% (Peery et al., 2015).

Multiple meta-analyses have indicated that surgical treatment yields the best therapeutic outcomes among various treatment methods for perianal diseases (Moult et al., 2015). Nevertheless, patients may encounter moderate to severe pain following perianal surgery (Medina-Gallardo et al., 2017), particularly during the initial dressing alteration or bowel movement (Sandler and Peery, 2019). Previous studies have reported that the incidence of postoperative urinary retention caused by perianal pain ranges from 3% to 50% (Kunitake and Poylin, 2016). Some acute severe pain after surgery can also affect the wound healing, extend the length of hospital stay, reduce the patients’ satisfaction (Zhang et al., 2022; Ni et al., 2023). Moreover, various data suggest that effective management of acute pain can reduce the risk of pain development (Sinatra, 2010; Gerbershagen et al., 2013). Therefore, it is crucial to explore effective postoperative analgesic methods for perianal surgery.

Clinically, various methods exist for analgesia in perianal surgery. Local infiltration blocks only relieve superficial pain (Borges et al., 2017). While caudal blocks provide effective pain relief, they have higher risks of motor block, intravascular injection (Kao and Lin, 2017), and postoperative urinary retention due to anatomical variations (Kim et al., 2005). Pudendal nerve blocks may offer significant postoperative analgesia.

The pudendal nerve is comprised of both sensory and motor nerves from the sacral plexus, originating from the S2-S4 spinal nerve roots. The pudendal nerve emits branches within the pudendal canal, which innervate the sensation and movement of the clitoris, penis, and perineum (Alkhaldi et al., 2015; Bonatti et al., 2022). Previous studies have shown that pudendal nerve block for postoperative analgesia provides a longer-lasting effect compared to epidural anesthesia, with a lower incidence of postoperative urinary retention and a higher benefit-to-risk ratio (Dadure et al., 2009; Ecoffey et al., 2010).

To extend the duration of local anesthetics and improve their effectiveness, adjuvants are often used in clinical practice (Hu et al., 2023). One such adjuvant is dexamethasone (DS), a long-acting glucocorticoid that has shown to prolong the duration of nerve blockade by reducing neuronal excitability and anti-inflammatory effects (Ribeiro et al., 2016).

Hence, this study intended to explore whether the combination of dexamethasone and ropivacaine can extend the duration of pudendal nerve block in patients undergoing perianal surgery under general anesthesia. The objective was to offer a safer and more effective postoperative analgesic regimen for patients with perianal diseases, facilitating their rapid recovery.

## 2 Patients and methods

### 2.1 Study design

The study was a single-center, double-blind, randomized controlled trial conducted at Chengdu Shangjin Nafu Hospital/West China Hospital, Sichuan University. This study had been approved by the Ethics Committee of Shangjin Nanfu Hospital (Date of approval: 20 March 2022. No. 2022032001) and registered in the Chinese Clinical Trial Registry (Registration No.: ChiCTR2100048760, Registration Date: 16 July 2021). Patients who agreed to participate in this study would be required to sign an informed consent form for the clinical trial.

### 2.2 Participants

The present study screened patients undergoing elective perianal surgery under general anesthesia in Shangjin Hospital, West China Hospital, Sichuan University from April 15 to 17 June 2022. The surgical types were hemorrhoids (Milligan-Morgan), anal fissure, anal fistula, and perianal abscess. The inclusion criteria were as follows: (Jain et al., 2019): patients undergoing elective anorectal surgery; (Peery et al., 2015); preoperative American Society of Anesthesiologists (ASA) classification of grade I-III; (Moult et al., 2015); patients aged between 18 and 65 years; (Medina-Gallardo et al., 2017); patients who agreed to participate in the study and required postoperative use of an analgesic pump.

The exclusion criteria were as follows: (Jain et al., 2019): participation in other clinical trials within the last 3 months; (Peery et al., 2015); patients with allergies or contraindications to the drugs used in this study; (Moult et al., 2015); patients with a history of chronic pain and long-term analgesic use before surgery; (Medina-Gallardo et al., 2017); Body Mass Index (BMI) ≥ 28 kg/m2 or ≤18 kg/m2; (Sandler and Peery, 2019); patients with communication difficulties.

### 2.3 Random and blind methods

The researchers grouped the patients in a 1:1 ratio using a random number table generated by Statistical Product and Service Solutions (SPSS) software. Based on the literature review and previous research data, the medication regimen for the P group in this study was as follows: unilateral 0.50% ropivacaine 10 mL, bilateral total of 20 mL. The medication regimen for the PD group was as follows: unilateral 0.50% ropivacaine 10 mL + 4 mg dexamethasone, bilateral total of 20 mL + 8 mg dexamethasone.

All participants, experimenters, and clinical doctors involved in this study were kept blind. In this study, all drugs used for pudendal nerve block would be prepared by a nurse who was unaware of the grouping and attached with random numbers on sealed treatment kits that were indistinguishable in appearance. After the subjects obtained random numbers, the physician responsible for the pudendal nerve block would open the corresponding packaged treatment kit. Data collection after the surgery would be carried out by personnel who were blind to the grouping.

### 2.4 Protocol

After the surgery, the patient was transferred to the Post-Anesthesia Care Unit (PACU) with an endotracheal tube. After the patient regained consciousness and met extubation criteria, the endotracheal tube was removed. Following the stabilization of vital signs, a pudendal nerve block was administered.

**FIGURE 1:**
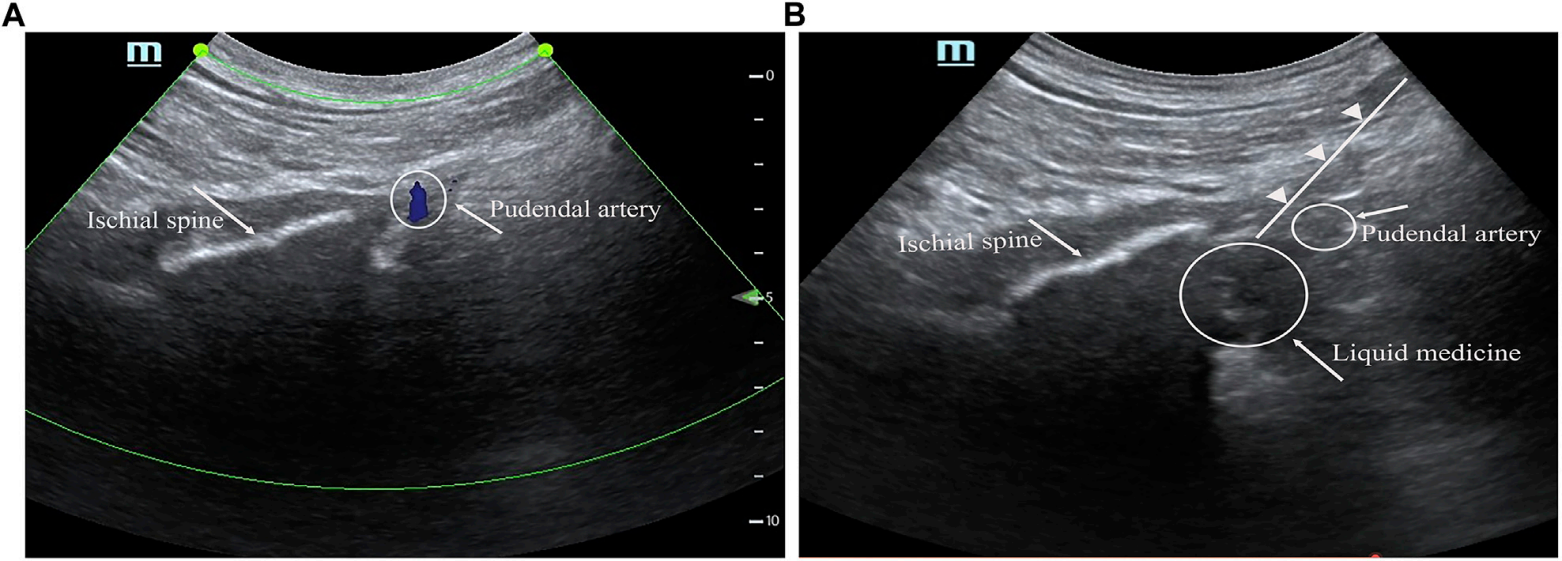
Ultrasound guided pudendal nerve block. **(A)**: Positioning image before injection **(B)**: image after injection. The white line represents the ischial spine, the smaller circle is the pudendal artery, the larger circle is the medicine, and the white triangle shows the needle’s trajectory.

The patient was advised to change from supine to prone position. Exposed both buttocks and sterilized the area with iodine solution, and strict aseptic technique was ensured. A 4.8 MHz phased array ultrasound probe was selected, positioned the marker towards the outer side of the body, placed the probe on one side of the buttocks, with the long axis perpendicular to the midpoint of the intergluteal cleft. At this point, the ultrasound image should reveal the internal ischial tuberosity and the external lesser trochanter of the femur. The ischial tuberosity was moved to the center of the screen, and then the probe was gradually moved toward the head until the ischial tuberosity disappeared and a long bright band of hyperintensity, known as the ischial spine, appeared (Figure 1A). The pudendal nerve was located between the tip of the ischial spine and the pudendal artery. Needle insertion was performed from the Mark point. After confirming the needle tip was in place and blood aspiration is negative, inject 1–2 mL of 5 ug/mL adrenaline. Once the medication was observed to spread around the pudendal nerve without significant changes in heart rate, proceed to inject the corresponding group of local anesthetic unilaterally, thus completing the pudendal nerve block (Figure 1B). Repeat the same procedure on the other side.

During the procedure, the patient was closely observed for any signs of local anesthetic toxicity, such as dizziness, tinnitus, palpitations, and changes in consciousness. After the anesthesia procedure was completed, the patient was placed in the supine position and vital signs were closely monitored. Once the PACU criteria were met, the patient would be transferred back to the ward.

### 2.5 Postoperative management

Postoperative pain management involved the utilization of Patient-Controlled Intravenous Analgesia (PCIA) by all patient. After the patient returned to the ward after surgery, the doctor administered 50 mg of flurbiprofen axetil twice a day. If the patient’s Numerical Rating Scale (NRS) score was ≥4, oral ketoprofen was given as rescue analgesia. If the patient’s NRS score was ≥7, intramuscular dezocine 5 mg was administered for pain relief and recorded. The NRS score was a pain level rating scale used to assess the intensity of a patient’s pain using a numerical method. A score of 0 indicated no pain, one to three indicated mild pain, four to six indicated moderate pain, and 7–10 indicated severe pain (Naja et al., 2005). The postoperative wound dressing of all patients included in this study consisted of Carbomer hemorrhoid gel and gauze block.

### 2.6 Sample size

In this study, we adopt the postoperative dressing for the first time the incidence of moderate to severe pain to calculate sample size. Based on the results of a preliminary experiment, the incidence of moderate to severe pain during the first dressing change was 53.8% in the P group and 33.3% in the PD group, the sample size required for this study was calculated to be n = 176 using Power Analysis and Sample Size (PASS) software. Considering a 10% dropout rate, a total of n = 194 patients were planned to be included in this trial.

### 2.7 Outcomes

The primary outcome of this study was the incidence of moderate to severe pain (NRS ≥4) during the first dressing change after surgery. A trained follow-up would ask the patient to record the intensity and nature of the most intense pain during the dressing change process.

The secondary outcomes included the resting and activity NRS scores at 2 h, 4 h, 6 h, 12 h, 24 h, 48 h, and 72 h after surgery. The activity NRS scores recorded the pain levels experienced by patients during activities such as turning and moving on the bed. Outcomes also included the incidence of moderate to severe pain during the first bowel movement after surgery, the rate of rescue analgesia within 72 h after surgery, the occurrence of perioperative adverse reactions (nausea, vomiting, urinary retention, lower limb numbness), QoR-15 scale within 3 days after surgery, sleep quality within 3 days after surgery (good, general, poor), and postoperative satisfaction level (great, good, general, poor). In the present study, patients who were unable to urinate while the bladder was full and needed to insert a catheter after surgery were considered to have postoperative urinary retention (Baldini et al., 2009).

The QoR-15 scale was used to evaluate the quality of patients’ early postoperative recovery. It consisted of a total of 15 items, each item was scored on a scale of 0–10, where 0 meant no presence and 10 meant always present. For negative indicators, the scoring was opposite. The sum of the scores was the patient’s QoR-15 related score (Kleif et al., 2018). Conclusions were drawn by comparing QoR-15 scores obtained using the same questionnaire within 3 days after surgery. Specific details about the QoR-15 scale could be found in Supplementary Material A.

### 2.8 Statistical analysis

Statistical analysis of the experimental data was conducted using SPSS 26.0 software. Normality of the data was assessed using the Kolmogorov-Smirnov test. For continuous variables with normal distribution, the variables were described using mean ± standard deviation, and independent samples *t*-test was used for between-group comparisons. If the variables were not normally distributed, they were described using median and interquartile range (IQR). The comparison of categorical variables was performed using either Pearson’s chi-square test or Fisher’s exact test, depending on the situation. A significance level of *p* < 0.05 was considered statistically significant.

According to previous research, a reduction of 1.1 in pain scores was considered the minimum clinically important difference (MCID) for pain intensity (Kelly, , 2001), and a decrease of at least 35% in the incidence of moderate to severe pain was considered the MCID (Sundarathiti et al., 2016; Meng et al., 2019). However, for QoR-15 scores, a change of at least 8 points was considered the MCID (Myles et al., 2016).

## 3 Result

**FIGURE 2:**
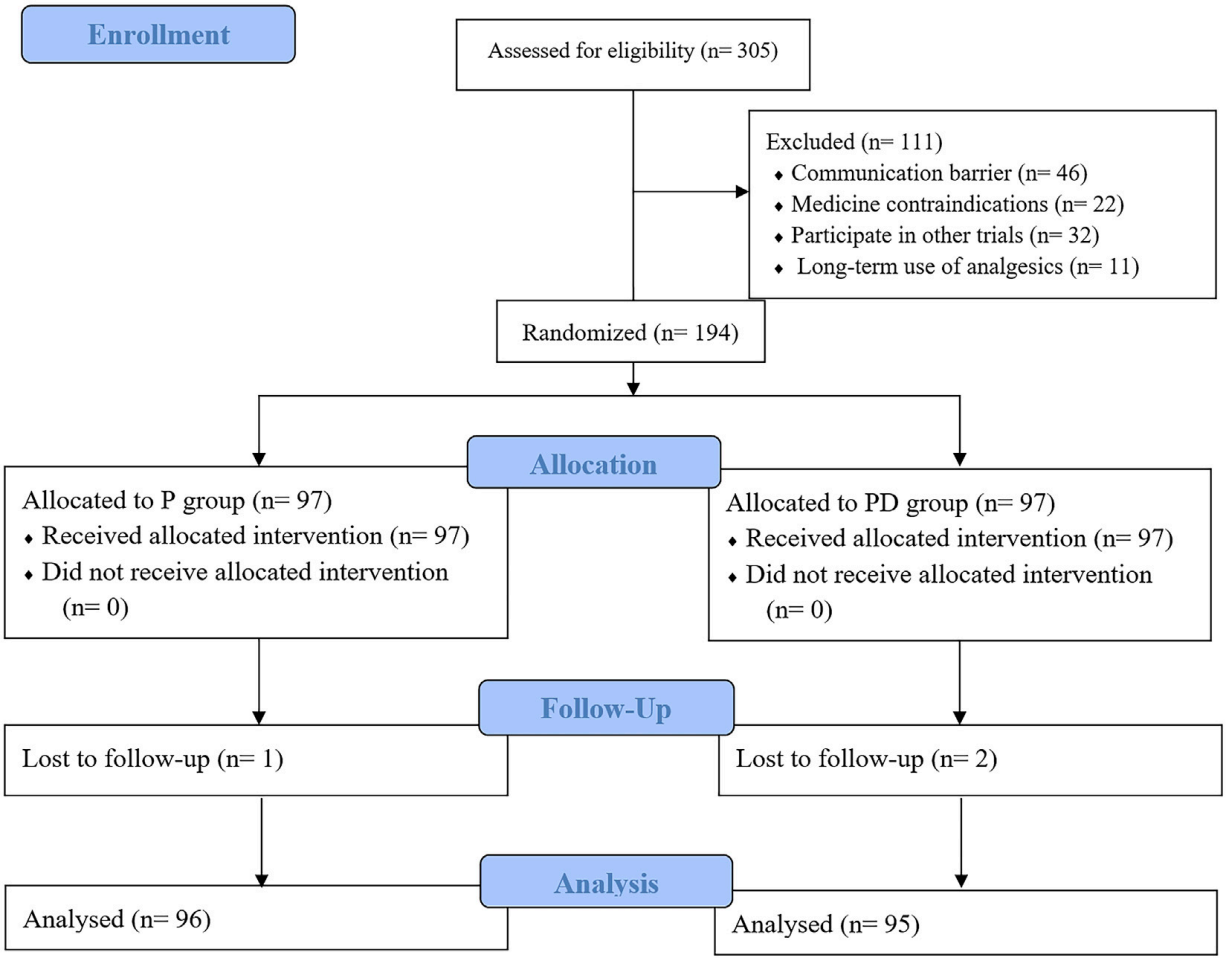
Flow chart of patient enrollment. The sample size is indicated in parentheses. P, pudendal nerve block; PD, pudendal nerve block + dexamethasone.

305 individuals who met the inclusion criteria were included in this study. A total of 111 individuals were excluded due to reasons such as communication barriers (n = 46), medicine contraindications (n = 22), participation in other trials (n = 32), and long-term use of analgesics (n = 11). Ultimately, 194 individuals were randomly assigned to the P group: PNB group (n = 97) and the PD group: PNB + DS group (n = 97). During the follow-up period, 3 individuals withdrew from the study for reasons such as patient refusal, resulting in 96 individuals included in the analysis for the P group and 95 individuals included in the analysis for the PD group (Figure 2).

The two groups showed no significant differences in baseline characteristics (age, sex, height, BMI, ASA classification, disease type, surgery time, anesthesia time, number of incisions, etc.), indicating comparability between the two groups (Table 1).

**TABLE 1:** Baseline demographic.

	P (n = 96)	PD (n = 95)	*p*-value
Age (years)	40.98 ± 10.89	41.96 ± 9.98	0.518^a^
Sex			0.943^b^
Male	49	48	
Female	47	47	
Height (cm)	165.77 ± 9.10	163.74 ± 8.01	0.105^a^
BMI (kg/m2)	22.06 ± 1.57	22.11 ± 1.49	0.825^a^
ASA			0.480^b^
I	14	15	
II	73	66	
III	9	14	
Diagnosis			0.719^b^
Mixed hemorrhoids	65	70	
Perianal abscess	9	6	
Anal fistula	19	15	
Anal fissure	3	4	
Duration of anesthesia (min)	56.98 ± 15.58	54.96 ± 16.79	0.390^a^
Duration of surgery (min)	33.06 ± 13.82	32.51 ± 14.79	0.788^a^
Number of incisions (n)	4.16 ± 3.39	4.39 ± 3.41	0.636^a^
Perianal dose (mL)	5.47 ± 2.52	5.81 ± 2.89	0.385^a^
Intraoperative infusion volume (mL)	251.04 ± 114.47	247.47 ± 107.36	0.824^a^

All values in the table represent the number or mean ± standard deviation.

BMI, body mass index; ASA, american society of anesthesiologists.

^a^Indicates that the *p*-value is derived from the Student’s t-test.

^b^ Indicates that the *p*-value is derived from the Pearson’s chi-squared test.

### 3.1 Primary outcome

**FIGURE 3:**
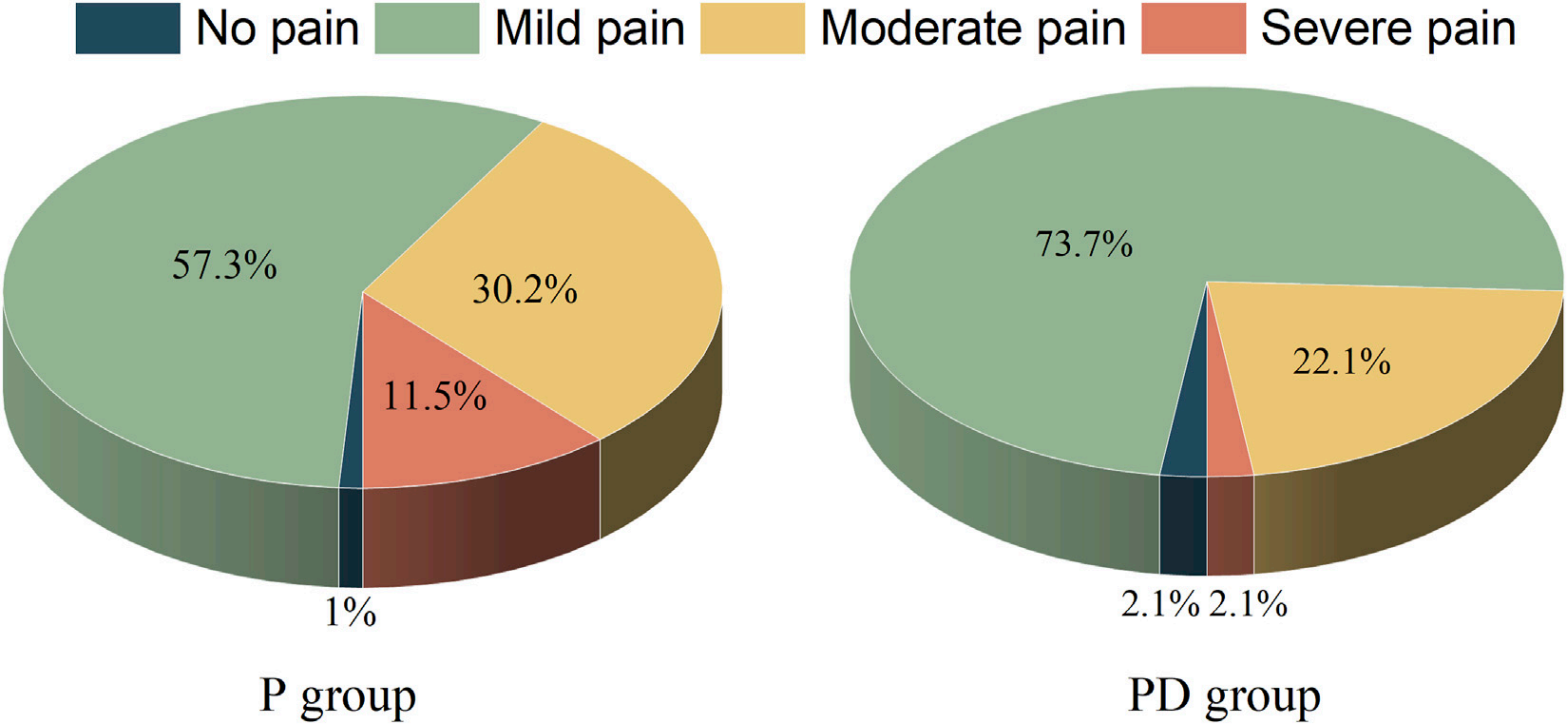
Proportion of pain during the first postoperative dressing change. The figure shows the percentage within the group. There were 96 participants in P group and 95 participants in PD group. P, pudendal nerve block; PD, pudendal nerve block + dexamethasone.

Regarding the occurrence rate of moderate to severe pain during the first dressing change after surgery (Figure 3), out of 96 patients in the P group, 40 experienced moderate to severe pain, with an incidence rate of 41.7%. In the PD group, out of 95 patients, 23 experienced moderate to severe pain, with an incidence rate of 24.2%. The incidence rate of moderate to severe pain during the first dressing change in the PD group decreased by 42% compared to the P group, with a *p*-value of 0.01, indicating the presence of statistical and clinical differences between the two groups.

### 3.2 Secondary outcomes

#### 3.2.1 Pain scores at each postoperative time point

Regardless of rest or movement, both groups showed an overall increasing trend in pain, with the highest level reached 24 h after surgery. Compared with group P, NRS scores in PD group at rest or movement at 2, 4, 6 and 12 h after surgery were statistically different. However, because the difference in NRS scores between the two groups was less than 1.1 points, the difference was not clinically significant (Supplementary Material B,C). No significant differences in NRS scores were observed between the two groups at 24, 48, and 72 h after surgery (Supplementary Material B,C).

#### 3.2.2 Postoperative recovery-related outcomes

**FIGURE 4:**
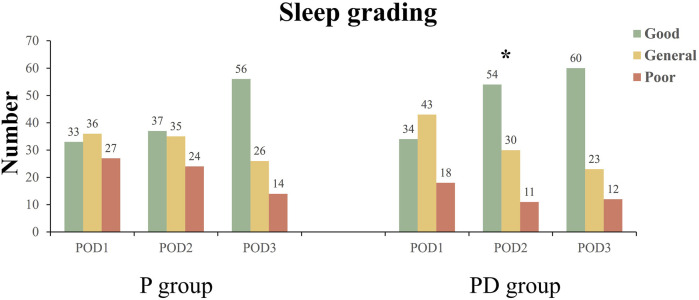
Postoperative sleep grading. The figure shows the sleep grading of the two groups 3 days after surgery. The number of people in each class is represented on the columns. * Significant at the 0.05 level and represents the comparison of sleep grading composition between the two groups. POD1: Post-Operative Day 1; P, pudendal nerve block; PD, pudendal nerve block + dexamethasone.

Within 3 days post-surgery, both groups’ QoR-15 scores increased. The PD group had significantly higher scores than the P group on Post-Operative Day 1 (POD1) and POD2 (*p* < 0.05, Table 2). However, on POD2, the P group had significantly more individuals with poor sleep compared to the PD group (Figure 4). Table 2 also shows the comparison of pain between the two groups during the first postoperative defecation.

**TABLE 2:** Postoperative recovery-related outcomes.

	P (n = 96)	PD (n = 95)	*p*-value
QoR-15, (score)			
POD1	138.09 ± 8.23	141.61 ± 7.48	0.002^a^
POD2	139.23 ± 6.67	144.97 ± 6.11	0.000^a^
POD3	144.05 ± 7.39	145.45 ± 6.13	0.156^a^
Time of first postoperative defecation, (h)	53.56 ± 11.65	53.31 ± 11.26	0.877^a^
Proportion of moderate to severe pain during defecation (%)	29 (30.2%)	14 (14.7%)	0.01^b^
Postoperative satisfaction			
Patient satisfaction^c^, (n)	50/20/22/4	69/13/12/1	0.023^b^
Medical satisfaction^c^, (n)	58/29/9/0	76/17/2/0	0.007^b^
Postoperative dosage of PCIA, (mL)	137.10 ± 16.48	123.59 ± 16.51	0.000^a^
Time of first postoperative discomfort, (h)	4.66 ± 5.03	8.21 ± 8.29	0.000^a^

All values in the table represent the number or mean ± standard deviation.

^a^Indicates that the *p*-value is derived from the Student’s t-test.

^b^ Indicates that the *p*-value is derived from the Pearson’s chi-squared test or Fisher’s test.

^c^ The satisfaction levels in the table are: great/good/general/poor.

Patients in the PD group experienced longer postoperative discomfort than those in the P group. However, both patients and medical staff reported significantly higher postoperative satisfaction in the PD group. Additionally, the PD group used significantly fewer analgesic pumps within the first 3 days after surgery compared to the P group.

#### 3.2.3 Comparison of adverse events between the two groups after surgery

Although data (Table 3) suggests that patients in the PD group may experience longer postoperative lower limb numbness, there were no significant differences between the two groups in the occurrence and severity of numbness. Similarly, there were no significant differences in postoperative nausea, vomiting, or rescue analgesia. However, the incidence of postoperative urinary retention was significantly lower in the PD group (2.1%) compared to the P group (11.5%, *p* = 0.01).

**TABLE 3:** Comparison of adverse events.

	P (n = 96)	PD (n = 95)	*p*-Value
Number of patients with postoperative urinary retention (%)	11 (11.5%)	2 (2.1%)	0.01^a^
Time of first postoperative urination, (h)	9.29 ± 4.39	9.43 ± 4.06	0.819^b^
The number of people with numbness in their lower limbs (%)	43 (44.8%)	51 (53.7%)	0.219^a^
Degree of numbness^c^	53/29/9/5	44/30/14/7	0.519^a^
Time for the numbness to disappear, (h)	5.05 ± 6.81	8.01 ± 8.62	0.009^b^
Number of people with PONV (%)	19 (19.8%)	16 (16.8%)	0.598^a^
Number of rescue analgesics (%)			
0–24 h	17 (17.7%)	21 (22.1%)	0.447^a^
24–48 h	16 (16.8%)	12 (12.6%)	0.431^a^
48–72 h	11 (11.5%)	9 (9.5%)	0.654^a^

All values in the table represent the number or mean ± standard deviation.

NRS: numerical rating scale.

PONV: postoperative nausea and vomiting.

^a^ Indicates that the *p*-value is derived from the Pearson’s chi-squared test or Fisher’s test.

^b^ Indicates that the *p*-value is derived from the Student’s t-test.

^c^ The classification of numbness in the table is: None/Acceptable/Normal/Discomfort.

## 4 Discussion

The study found for the first time that the use of dexamethasone in conjunction with ropivacaine, a frequently used local anesthetic used for pudendal nerve block, could be an effective method of reducing the incidence of moderate to severe pain during the first dressing change (48 h postoperatively) and the first bowel movement after surgery. Additionally, the combination of medications may contribute to a speedier postoperative recovery, improve postoperative sleep quality and overall patient satisfaction levels, and lower the occurrence of postoperative urinary retention. For Chinese patients who need hospitalization, the perioperative quality of life of patients has been greatly improved.

In this study, we opted to administer the pudendal nerve block in the PACU while patients were awake, rather than preoperatively following the induction of anesthesia. The rationale behind this decision was twofold: firstly, performing the nerve block postoperatively would not interfere with the surgical procedure; secondly, administering the block while the patient was conscious would enable the physician to more accurately monitor the patient’s response, thereby minimizing the risk of overlooking potential block-related adverse effects.

Because most patients after perianal surgery experience the most severe pain during the first dressing change and first bowel movement throughout the entire hospitalization process, the pain intensity was comparable to that of some major surgeries, and pain cannot be effectively relieved by PCIA or oral medications (Simillis et al., 2015; Medina-Gallardo et al., 2017). Since the time of the patient’s first bowel movement after surgery could not be accurately predicted and there may be recall bias at follow-up, the primary outcome of this study was set as pain at the first dressing change. In our hospital, the first dressing change for patients was scheduled on the second day after surgery, during which the incision is disinfected and dressed with pressure, and the whole process takes approximately 10 min. However, previous studies had reported (Di Giuseppe et al., 2020; He et al., 2021) that with the use of 0.5%–0.75% ropivacaine or bupivacaine for pudendal nerve block, only pain within 24 h after surgery can be relieved, and the stimulation during the dressing change on the second day after surgery caused the postoperative pain to peak. The results of this study also demonstrated that the proportion of patients in the PD group experiencing moderate to severe pain during the dressing change on the second day after surgery was lower than that in the P group, and the pain was less intense. Thus, adding adjuvants to the local anesthetic might prolong the duration of postoperative analgesia and alleviated the pain during the first dressing change for patients.

In terms of secondary outcomes, the assessment of sleep quality depended on the relief of pain and the influence of the surrounding environment (Ljungqvist et al., 2017). In the PD group, the use of dexamethasone may prolong the analgesic duration, thereby improving the sleep quality of patients on the second day after surgery (Albrecht et al., 2015; Baeriswyl et al., 2017). This outcome indicator suggested that the addition of dexamethasone to ropivacaine can improve the sleep quality of patients on the second night after surgery, which may be related to the reduction of discomfort in patients that night (Labat et al., 2017).

In terms of adverse event-related outcome, we focused on sensory blockade and motor blockade in two groups of patients. In terms of motor blockade, the study found that dexamethasone did not increase the time for patients to get out of bed and the time for bowel movement after surgery. In the context of sensory blockade, patients in the PD group gradually experienced the first discomfort of pain after approximately 8 h postoperatively, which was significantly different from the P group. However, nearly half of the patients experienced an increase in the duration of postoperative lower limb numbness. Despite an increase in the duration of lower limb numbness by approximately 3 h, it was found that most patients could tolerate the current level of numbness and did not experience significant discomfort when assessing its severity statistically. This was also evident from the perspective of patient satisfaction.

The occurrence rate of urinary retention was the most significant adverse outcome measure in this study. Regional blockade or other nerve blocks were commonly used for postoperative analgesia in previous studies on perianal surgery. Although most methods can provide patients with a satisfactory postoperative analgesic experience, further development was restricted due to the higher occurrence rate of postoperative urinary retention.

In this study, the incidence of urinary retention in the P group was consistent with previous studies, however, in the PD group where dexamethasone was added, the postoperative urinary retention rate was only 2.1%, significantly lower than the 11.5% in the P group (Hong et al., 2017; Denham et al., 2019). It was revealed that adding dexamethasone to local anesthetic ropivacaine for nerve blockade can prolong the analgesic effect and reduce the occurrence of postoperative urinary retention. The possible mechanisms were might related to regulating the excitability of nociceptive neurons, reducing the release of inflammatory mediators, inhibiting the surgical-induced inflammatory response, providing effective analgesic effects etc (Hough et al., 2003; Sacco et al., 2003).

The present study had the following limitations: firstly, the result of this study indicate that dexamethasone can effectively alleviate moderate to severe pain experienced during the first dressing change after surgery, but the optimal dosage of the dexamethasone for prolonging the analgesic duration of pudendal nerve block remained to be explored. Second, due to the use of multimodal analgesia in this study, the NRS scores of the patients were relatively low, which might have reduced the differences between the PD and P groups. In addition to the prescribed post-operative pain management strategy, we did not restrict the use of other drugs, such as hypnotics, which may have an impact on recovery quality, sleep grading and other results. Additionally, there may be recall bias during the follow-up process of this study, which could potentially affect the outcome measures of this study. Finally, the surgical procedures encompassed in this study included mixed hemorrhoids, perianal abscesses, anal fistulas, and anal fissures. When interpreting the study results, proctological procedures was mixed together in the analysis, which could have skewed the results. Different types of surgery may lead to different degrees of pain experience, and local postoperative care is also different. For example, the pain after anal fistula surgery is different from that after tripedicular haemorrhoidectomy. Furthermore, the surgeries were not uniformly performed by a single surgeon, introducing potential variability in surgical techniques and procedures. This variability among surgeons could contribute to differences in postoperative patient experiences, thereby influencing the study outcomes.

## 5 Conclusion

The study found that adding dexamethasone to ropivacaine in the pudendal nerve block can reduce the incidence of moderate to severe pain during the first dressing change, improve sleep quality, reduce analgesic drug dosage, and the patients in the PD group did not experience additional adverse reactions except for a longer duration of numbness, promoting rapid postoperative recovery of the patients.

## Data Availability

The raw data supporting the conclusion of this article will be made available by the authors, without undue reservation.
