# Supplementary material for: The impact of combined administration of ropivacaine and dexamethasone on postoperative analgesia in perianal surgery with pudendal nerve block under ultrasound guidance: a prospective randomized controlled study
Source: Front Pharmacol. 2024 Jun 27;15:1366070. doi: 10.3389/fphar.2024.1366070 (PMC11236761; doi:10.3389/fphar.2024.1366070)
Supplement: Supplementary file 1 [file DataSheet2.DOC]

**
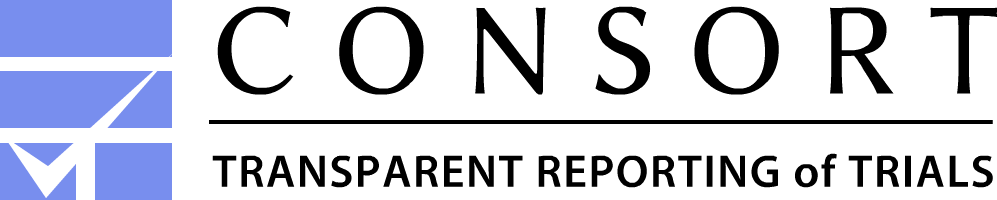
**

**CONSORT 2010 Flow Diagram**

**Allocation**

**Follow-Up**

**Enrollment**

Assessed for eligibility (n= 305)

Excluded (n= 111)

 ♦ Communication barrier (n= 46)

 ♦ Medicine contraindications (n= 22)

♦ Participate in other trials (n= 32)

♦ Long-term use of analgesics (n= 11)

Allocated to P group (n= 97)

♦ Received allocated intervention (n= 97)

♦ Did not receive allocated intervention (n= 0)

Allocated to PD group (n= 97)

♦ Received allocated intervention (n= 97)

♦ Did not receive allocated intervention (n= 0)

Randomized (n= 194)

Lost to follow-up (n= 1)

Lost to follow-up (n= 2)

**Analysis**

Analysed (n= 95)

Analysed (n= 96)
