# Supplementary material for: The impact of combined administration of ropivacaine and dexamethasone on postoperative analgesia in perianal surgery with pudendal nerve block under ultrasound guidance: a prospective randomized controlled study
Source: Front Pharmacol. 2024 Jun 27;15:1366070. doi: 10.3389/fphar.2024.1366070 (PMC11236761; doi:10.3389/fphar.2024.1366070)
Supplement: Supplementary file 2 [file DataSheet1.DOCX]

Supplementary Material

# Supplementary Data

Supplementary A QoR-15 Scale

**Name：_____________ Telephone：_______________**

| How have you been feeling in the last 24 hours?  0 to 10, 0 = none of the time (poor) and 10 = all of the time (excellent) | |
| --- | --- |
| 1. Able to breathe easily | 0 1 2 3 4 5 6 7 8 9 10 |
| 2. Been able to enjoy food | 0 1 2 3 4 5 6 7 8 9 10 |
| 3. Feeling rested | 0 1 2 3 4 5 6 7 8 9 10 |
| 4. Have had a good sleep | 0 1 2 3 4 5 6 7 8 9 10 |
| 5. Able to look after personal toilet and hygiene unaided | 0 1 2 3 4 5 6 7 8 9 10 |
| 6. Able to communicate with family or friends | 0 1 2 3 4 5 6 7 8 9 10 |
| 7. Getting support from hospital doctors and nurses | 0 1 2 3 4 5 6 7 8 9 10 |
| 8. Able to return to work or usual home activities | 0 1 2 3 4 5 6 7 8 9 10 |
| 9. Feeling comfortable and in control | 0 1 2 3 4 5 6 7 8 9 10 |
| 10. Having a feeling of general well-being | 0 1 2 3 4 5 6 7 8 9 10 |
| Have you had any of the following in the last 24 hours?  10 to 0, 10 = none of the time (excellent) and 0 = all of the time (poor) | |
| 11. Moderate pain | 10 9 8 7 6 5 4 3 2 1 0 |
| 12. Severe pain | 10 9 8 7 6 5 4 3 2 1 0 |
| 13. Nausea or vomiting | 10 9 8 7 6 5 4 3 2 1 0 |
| 14. Feeling worried or anxious | 10 9 8 7 6 5 4 3 2 1 0 |
| 15. Feeling sad or depressed | 10 9 8 7 6 5 4 3 2 1 0 |

# Supplementary Tables

Table B. NRS score at each time (resting)

|  | **P (n = 96)** | **PD (n = 95)** | ***P* Value** |
| --- | --- | --- | --- |
| NRS |  |  |  |
| 2h | 0[0 - 1] | 0[0 - 1] | 0.001 |
| 4h | 1[0 - 1] | 0[0 - 1] | 0.003 |
| 6h | 1[0 - 1] | 0[0 - 1] | 0.001 |
| 12h | 1[0 - 2] | 0[0 - 1] | 0.000 |
| 24h | 1[0 - 2] | 1[0 - 2] | 0.130 |
| 48h | 1[0 - 2] | 0[0 - 1] | 0.055 |
| 72h | 1[0 - 2] | 1[0 - 2] | 0.073 |

All values in the table represent the median [IQR]

The P-values in the table are derived from Mann-Whitney U test

Table C. NRS score at each time (moving)

|  | **P (n = 96)** | **PD (n = 95)** | ***P* Value** |
| --- | --- | --- | --- |
| NRS |  |  |  |
| 2h | 1[1 - 2] | 1[1 - 1] | 0.000 |
| 4h | 2[1 - 2] | 1[1 - 2] | 0.022 |
| 6h | 2[1 - 2.75] | 1[1 - 2] | 0.000 |
| 12h | 2[1 - 3] | 1[1 - 2] | 0.000 |
| 24h | 2[1 - 3] | 2[1 - 3] | 0.202 |
| 48h | 2[1 - 3] | 1[1 - 3] | 0.135 |
| 72h | 2[1 - 3] | 2[1 - 3] | 0.111 |

All values in the table represent the median [IQR]

The P-values in the table are derived from Mann-Whitney U test
